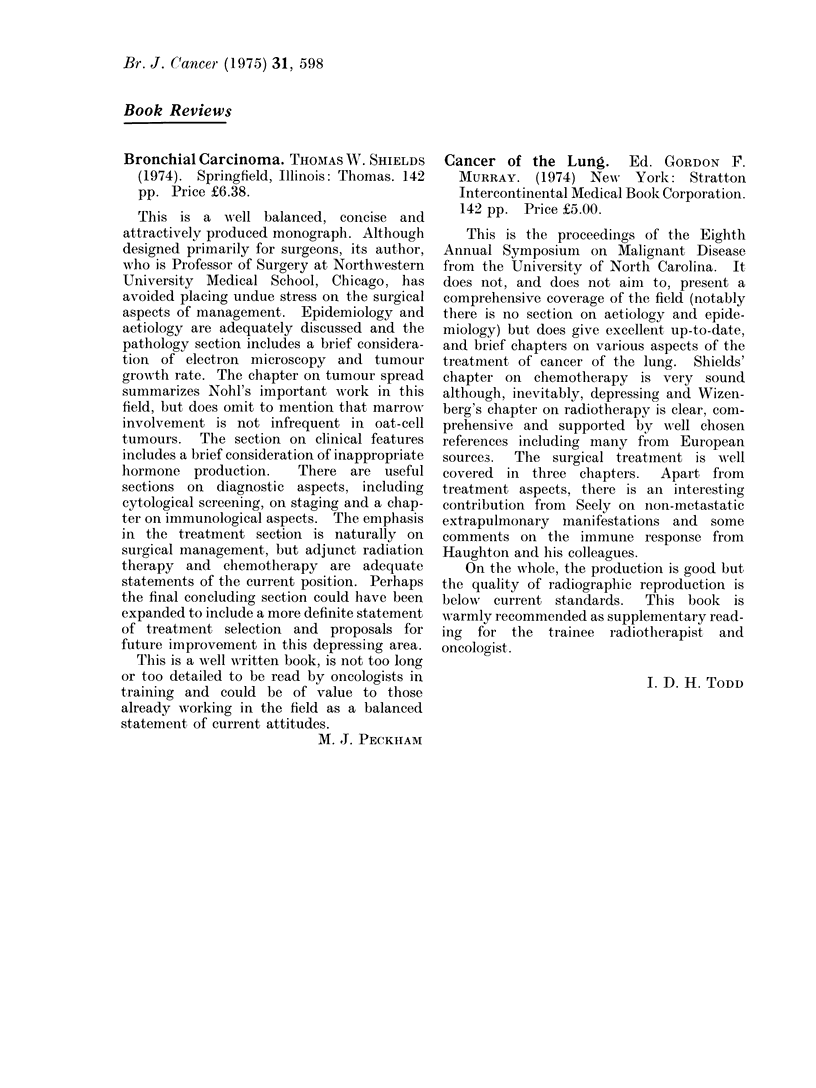# Bronchial Carcinoma

**Published:** 1975-05

**Authors:** M. J. Peckham


*Br. J. Cancer* (1975) **31**, 598

### *Book Reviews*

**Bronchial Carcinoma.** THOMAS W. SHIELDS (1974). Springfield, Illinois: Thomas. 142 pp. Price £6.38.

This is a well balanced, concise and attractively produced monograph. Although designed primarily for surgeons, its author, who is Professor of Surgery at Northwestern University Medical School, Chicago, has avoided placing undue stress on the surgical aspects of management. Epidemiology and aetiology are adequately discussed and the pathology section includes a brief consideration of electron microscopy and tumour growth rate. The chapter on tumour spread summarizes Nohl's important work in this field, but does omit to mention that marrow involvement is not infrequent in oat-cell tumours. The section on clinical features includes a brief consideration of inappropriate hormone production. There are useful sections on diagnostic aspects, including cytological screening, on staging and a chapter on immunological aspects. The emphasis in the treatment section is naturally on surgical management, but adjunct radiation therapy and chemotherapy are adequate statements of the current position. Perhaps the final concluding section could have been expanded to include a more definite statement of treatment selection and proposals for future improvement in this depressing area.

This is a well written book, is not too long or too detailed to be read by oncologists in training and could be of value to those already working in the field as a balanced statement of current attitudes.

M. J. PECKHAM